# Supplementary material for: scCobra allows contrastive cell embedding learning with domain adaptation for single cell data integration and harmonization
Source: Commun Biol. 2025 Feb 13;8:233. doi: 10.1038/s42003-025-07692-x (PMC11825689; doi:10.1038/s42003-025-07692-x)
Supplement: Supplementary file 1 — Supplementary Information [file 42003_2025_7692_MOESM1_ESM.pdf]

scCobra allows contrastive cell embedding learning with domain adaptation for single cell data integration and harmonization

Bowen Zhao[1,2,3], Kailu Song[2, 4], Dong-Qing Wei[1], Yi Xiong[1*], and Jun Ding[2, 3, 4, 5, 6*]

[1]State Key Laboratory of Microbial Metabolism, School of Life Sciences and Biotechnology, Shanghai Jiao Tong University, Shanghai, 200240, China

[2]Meakins-Christie Laboratories, Department of Medicine, McGill University Health Centre, Montreal, QC, H4A 3J1, Canada

[3]Medicine, Division of Experimental Medicine, McGill University, 1001 Decarie Blvd, Montreal H4A 3J1 Quebec, Canada

[4]Quantitative Life Sciences, McGill University, Montreal, QC, Canada.

[5]School of Computer Science, McGill University, 3480 Rue University, Montreal, H3A 2A7, Quebec, Canada.

[6]Mila-Quebec AI Institute, 6666 Rue Saint-Urbain, Montreal, H2S3H1, Quebec, Canada.

[*]All correspondences should be addressed to Y.X (xiongyi@sjtu.edu.cn) and J.D (jun.ding@mcgill.ca)

Supplementary Figures

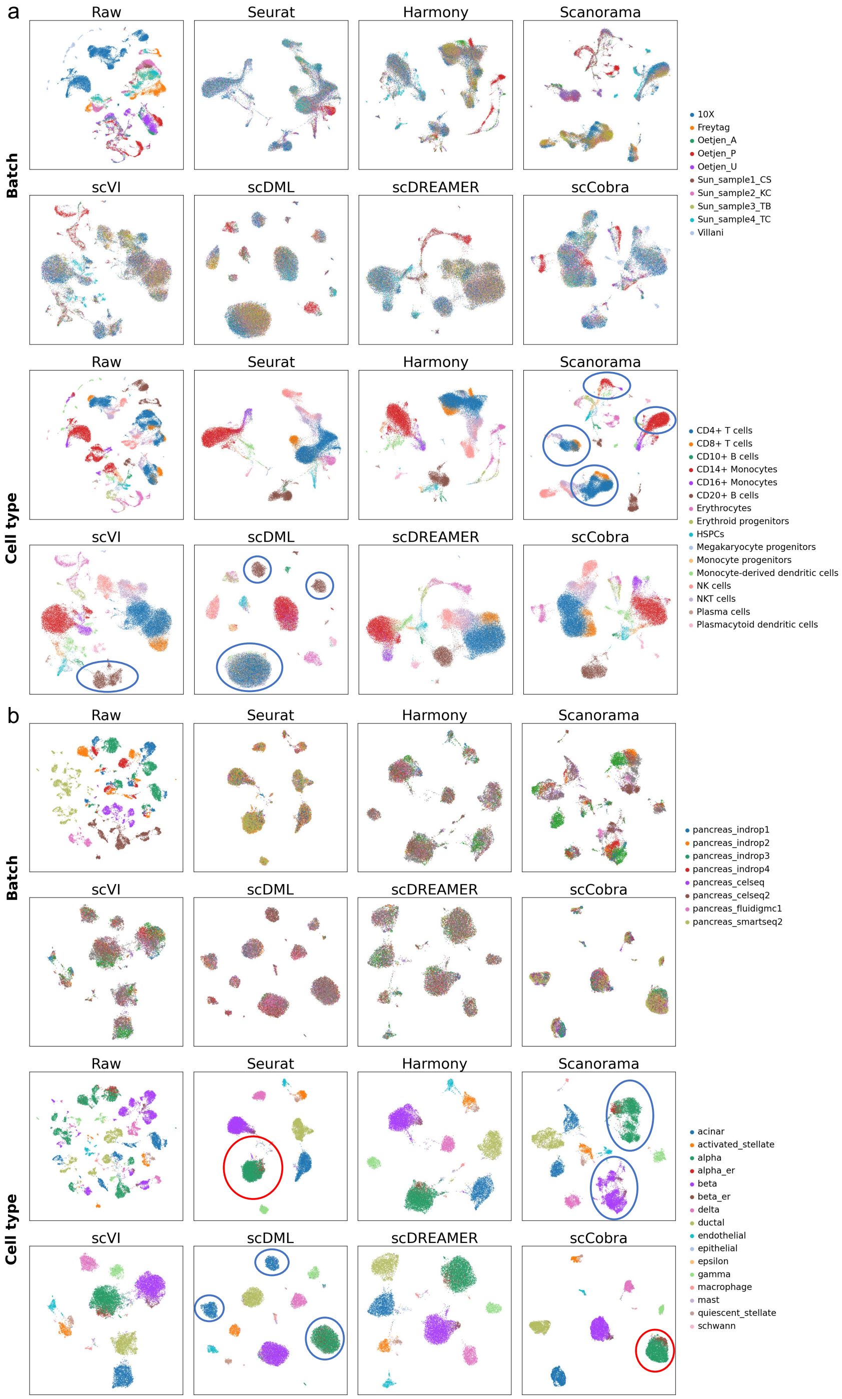

Fig. S1 scCobra demonstrates superior batch correction performance over state-of-the-art methods. a, UMAP visualization comparing scCobra's performance against benchmarked methods (Seurat, Harmony, Scanorama, scVI, scDML and scDREAMER) on the immune dataset. The visualization is split into four rows: the top two rows show batch correction results, with different batches distinguished by color, and the bottom two rows present cell type aggregation, with each cell type assigned a unique color. **b,** UMAP visualization comparing scCobra's performance against existing methods (Seurat, Harmony, Scanorama, scVI, scDML and scDREAMER) on the pancreas dataset. The visualization is split into four rows: the top two rows show batch correction results,

with different batches distinguished by color, and the bottom two rows present cell type aggregation, with each cell type assigned a unique color.

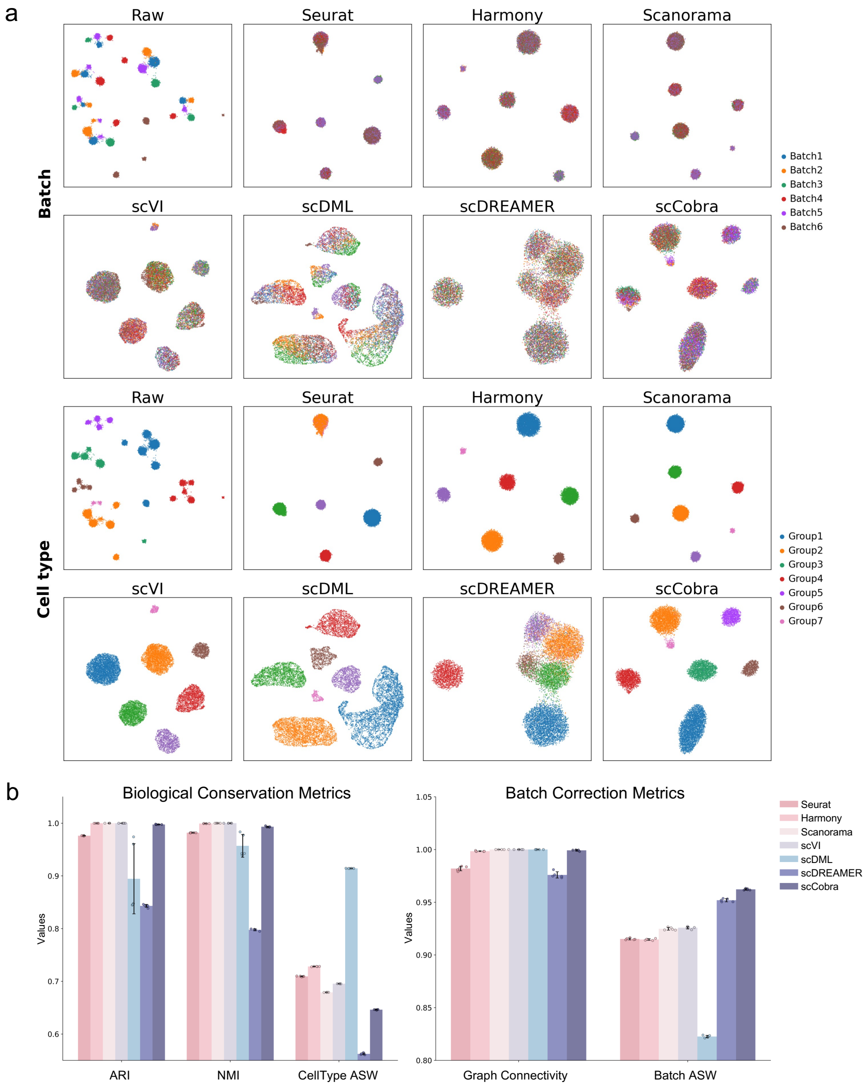

Fig. S2 scCobra demonstrates superior batch correction performance over state-of-the-art methods. a, UMAP visualization comparing scCobra's performance against benchmarked methods (Seurat, Harmony, Scanorama, scVI, scDML and scDREAMER) on the simulation dataset. The visualization is split into four rows: the top two rows show batch correction results, with different batches distinguished by color, and the bottom two rows present cell type aggregation, with each cell type assigned a unique color. **b,** Quantitative results of scCobra and the benchmarked methods on simulation dataset (n=5 independent experiments). We provide two types of evaluation metrics. Biological conservation metrics include ARI, NMI, and CellType ASW, with higher values indicating better retention of biological signals during batch effect removal. Batch correction metrics include Graph Connectivity and Batch ASW, where higher values signify better mixing of cells from different batches and more effective removal of batch effects. This panel differentiates the performance of each method using color coding, providing a clear comparison of their effectiveness in addressing batch effects and enhancing data comparability. Error bars represent the standard deviation of the evaluated metrics across datasets.

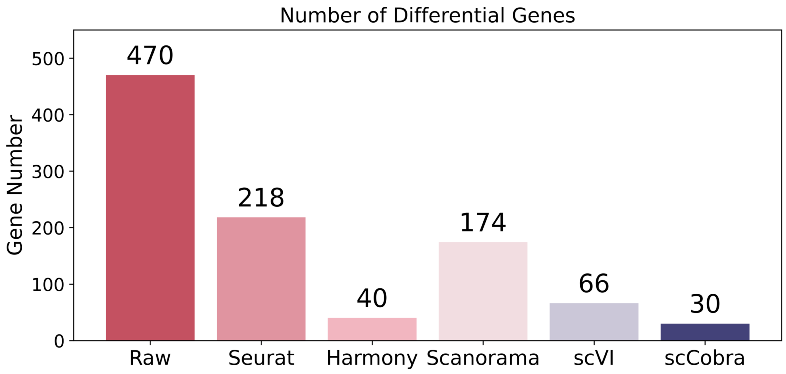

Fig. S3 scCobra effectively eliminates spurious differential signals caused by batch effects. The figure shows the differences in the number of differentially expressed genes (DEGs) between samples under the same condition, comparing raw gene expression with corrected gene expression using scCobra and other methods. The results demonstrate that scCobra significantly reduces the number of DEGs driven by batch effects, outperforming other methods in eliminating batch-induced noise.

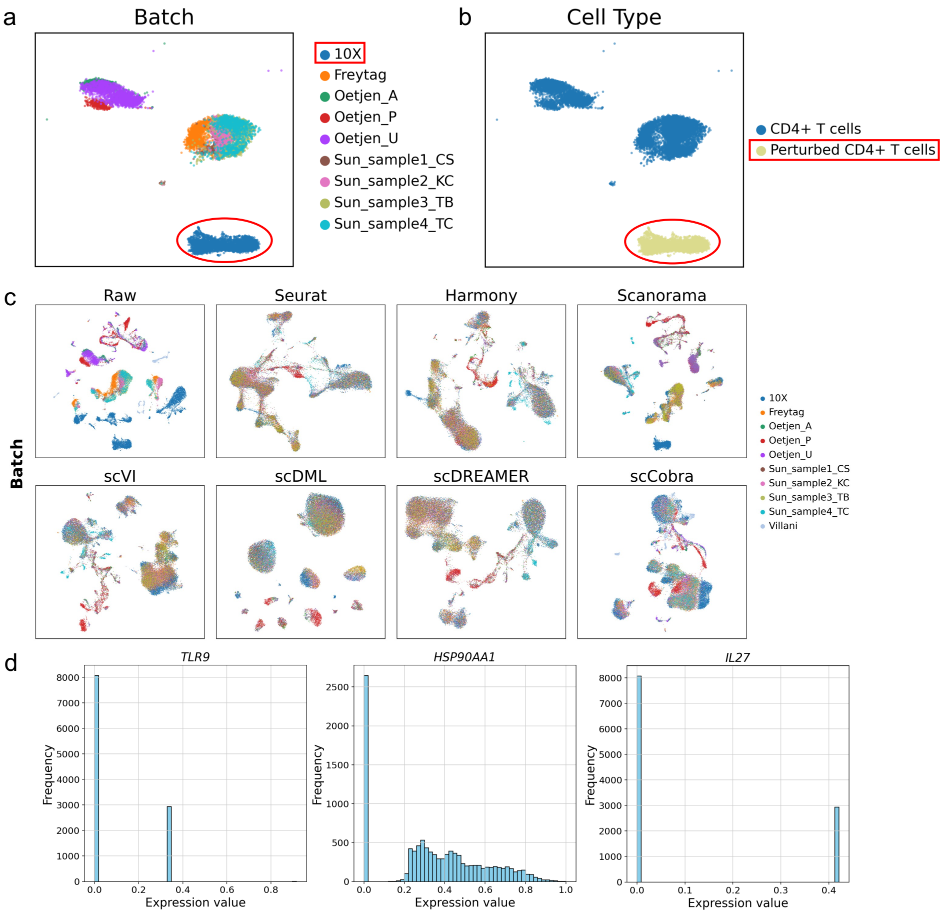

Fig. S4 scCobra is capable of distinguishing perturbed *CD4+* T cells from their unperturbed counterparts. a, UMAP visualization of the immune dataset, showing perturbed *CD4+* T cells and *CD4+* T cells, colored by batch. **b**, UMAP visualization of the same immune dataset, displaying perturbed *CD4+* T cells and *CD4+* T cells, colored by cell type. **c**, Batch correction methods (Seurat, Harmony, Scanorama, scVI, scDML, scDREAMER, and scCobra) were assessed on a simulated immune scRNA-seq dataset with batch effects and biological differences introduced by perturbing genes associated with the " Reactome influenza life cycle " Reactome pathway. The UMAP visualization is colored by batch.

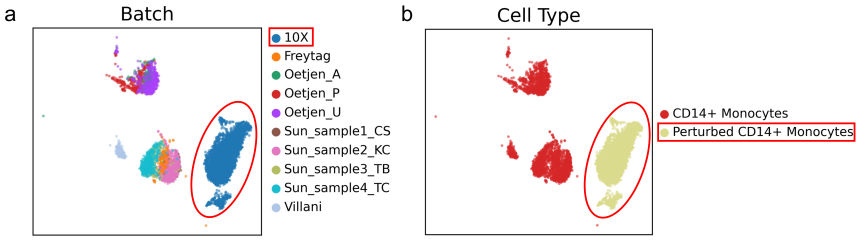

Fig. S5 UMAP visualization of *CD14*+ Monocytes and Perturbed *CD14*+ Monocytes from the simulated immune dataset. **a**, UMAP visualization of the simulated immune dataset, showing perturbed *CD14*+ Monocytes and *CD14*+ Monocytes, colored by batch. **b**, UMAP visualization of the same simulated immune dataset, displaying perturbed *CD14*+ Monocytes and *CD14*+ Monocytes, colored by cell type.

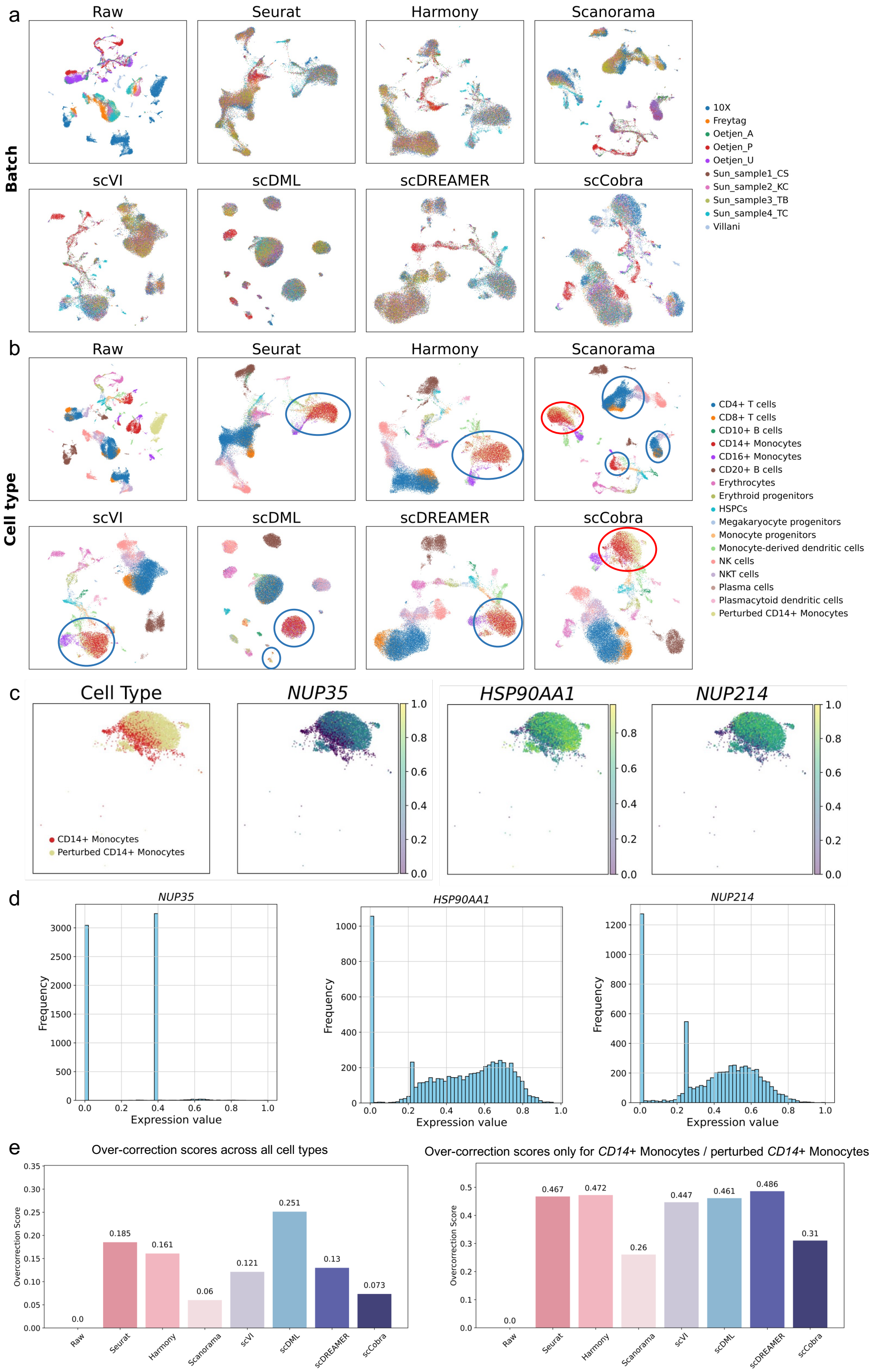

Fig. S6 scCobra is capable of distinguishing perturbed *CD14*+ Monocytes from their unperturbed counterparts. a, b, Batch correction methods (Seurat, Harmony, Scanorama, scVI, scDML, scDREAMER, and scCobra) were assessed on a simulated immune scRNA-seq dataset with batch effects and biological differences introduced by perturbing genes associated with the "

Reactome influenza life cycle " Reactome pathway. The UMAP visualization is colored by batch (a) and cell type (b). The results demonstrate that scCobra effectively distinguishes perturbed *CD14*+ Monocytes from their unperturbed counterparts during batch integration, with significantly lower levels of over-correction compared to other methods. **c**, Enlarged UMAP plots clearly show that scCobra effectively separates normal *CD14*+ Monocytes from perturbed *CD14*+ Monocytes. This distinction is highlighted by specific markers such as *NUP35*, *HSP90AA1*, *NUP214* demonstrating that differential markers can reliably distinguish perturbed cells from normal cells. This figure underscores scCobra's ability to maintain critical biological variation after batch correction. **d,** The x-axis represents gene expression levels, while the y-axis indicates the frequency of cells expressing the corresponding levels. This figure demonstrates that not all genes exhibit binary expression patterns, with some showing a range of expression levels across cells. **e**, Quantitative over-correction analysis on all cell types and specifically for *CD14*+ Monocytes and perturbed *CD14*+ Monocytes. Higher values indicate a greater degree of over-correction, whereas values closer to zero reflect minimal over-correction. The results demonstrate that scCobra significantly reduces the risk of over-correction compared to existing methods, as indicated by its lower over-correction scores.

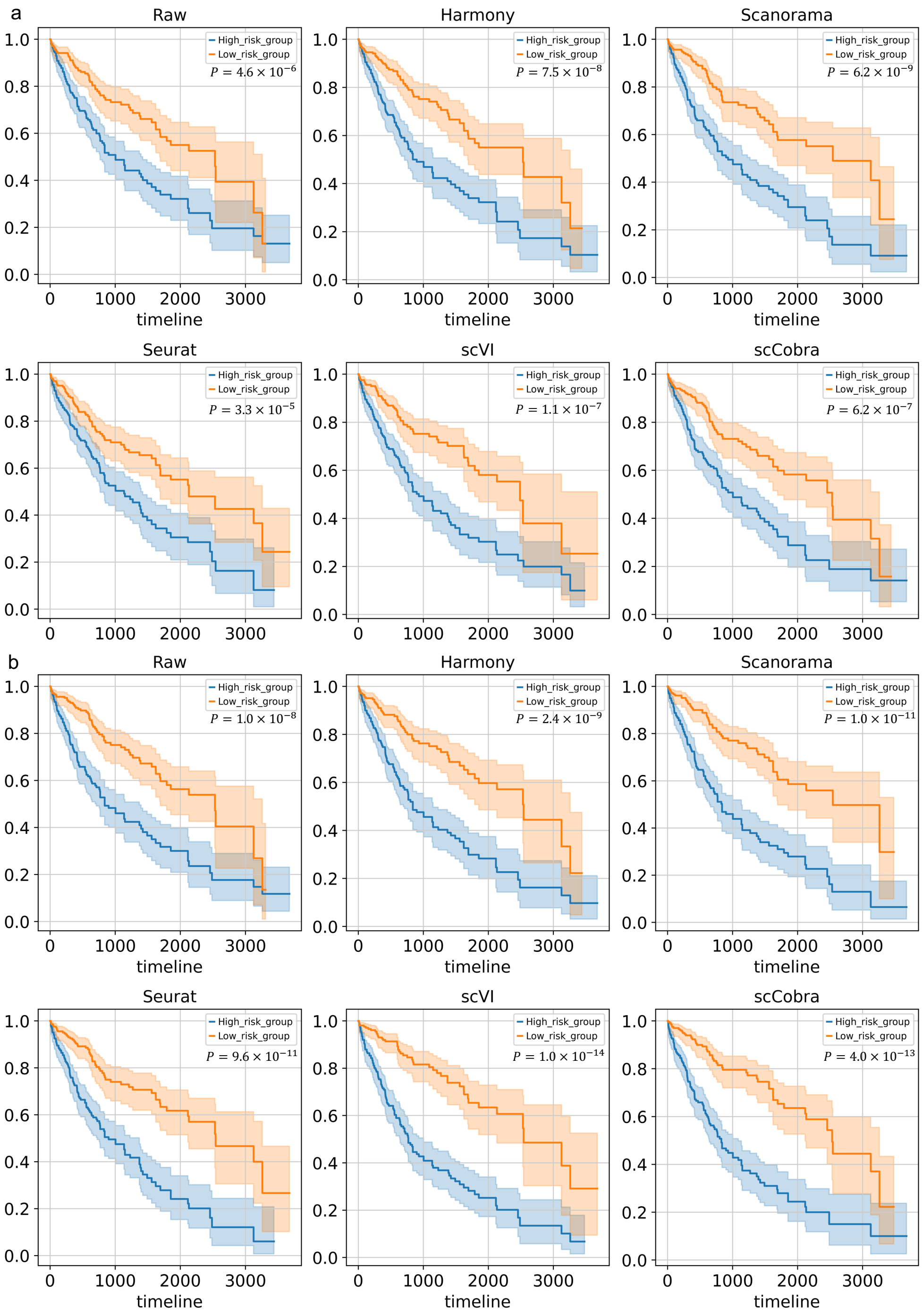

Figure S7 Survival analysis results using different cut-off thresholds for top-ranked differential gene sets. Survival analysis was performed using differential gene sets obtained from corrected gene expression with scCobra and benchmarking methods. Blue curves represent high-risk groups, and orange curves represent low-risk groups, with the x-axis indicating survival days and the y-axis representing the survival proportion. The analysis demonstrates that scCobra-corrected gene expression effectively eliminates batch noise and identifies meaningful differential genes that stratify patients into distinct risk groups, improving survival classification compared to raw and other corrected gene expression methods. Results using the top 25 differential genes are shown in (**a**), while results using the top 50 differential genes are shown in (**b**).

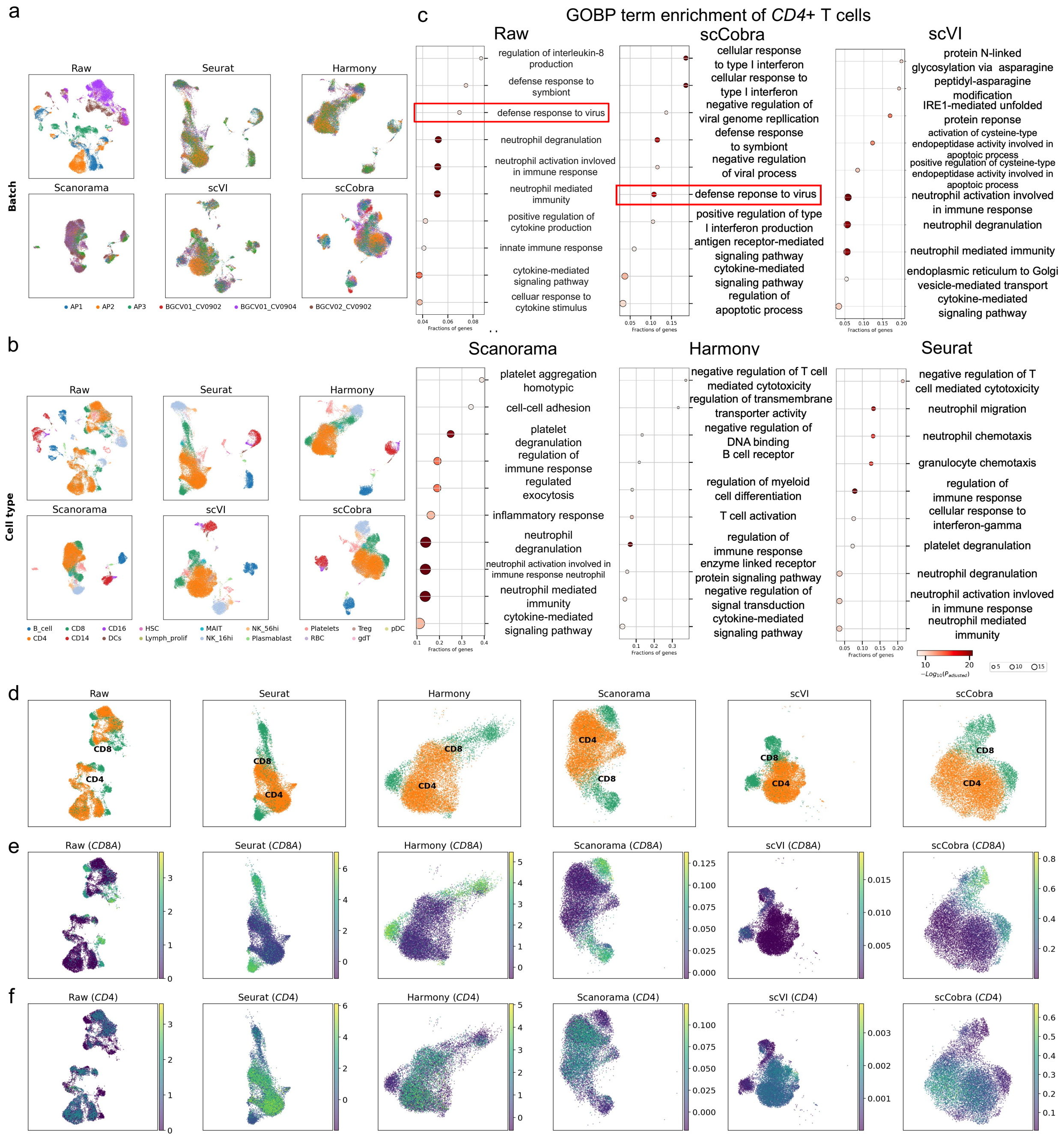

Fig. S8 scCobra Can Minimize Over-correction Risk. a, b, Batch correction methods (Seurat, Harmony, Scanorama, scVI and scCobra) were assessed on the COVID-19 dataset. The UMAP visualization is colored by batch (**a**) and cell type (**b**). **c,** Post-correction Gene Ontology enrichment analysis for the COVID-19 dataset underscores scCobra's capability to retain the "defense response to virus" GOBP term, which is a top-identified term in the raw (uncorrected) COVID data, crucial for COVID sample analysis. This stands in contrast to other methods, which exhibit correction inaccuracies, evidenced by the loss of this critical GOBP term, indicative of over-correction. **d**, Enlarged UMAP plots demonstrate that *CD4*+ and *CD8*+ T cells are clearly separated, with UMAPs colored by cell type. **e, f,** Specific markers, such as *CD4* for *CD4*+ T cells and *CD8A* for *CD8*+ T cells, effectively identify and distinguish these two cell types. These results show that scCobra preserves the distinct identities of *CD4*+ and *CD8*+ T cells without mixing, avoiding over-correction.

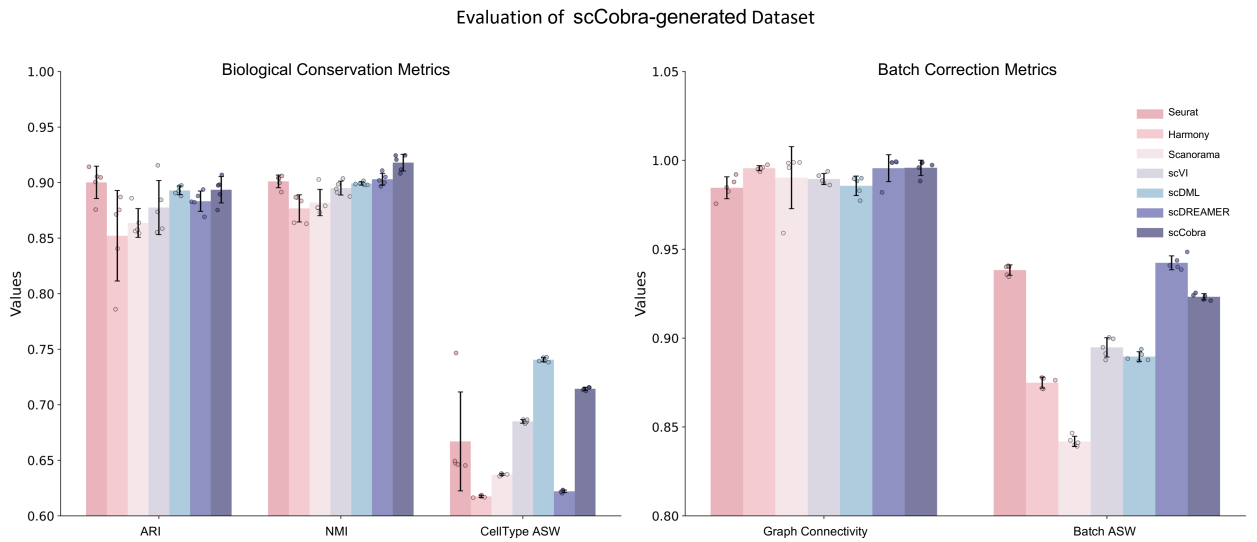

Figure S9 The evaluation of multiple methods benchmarked on scCobra-generated dataset. Quantitative results of scCobra and the benchmarked methods on scCobra-generated dataset (n=5 independent experiments). We provide two types of evaluation metrics. Biological conservation metrics include ARI, NMI, and CellType ASW, with higher values indicating better retention of biological signals during batch effect removal. Batch correction metrics include Graph connectivity and Batch ASW, where higher values signify better mixing of cells from different batches and more effective removal of batch effects. This panel differentiates the performance of each method using color coding, providing a clear comparison of their effectiveness in addressing batch effects and enhancing data comparability. Error bars represent the standard deviation of the evaluated metrics across datasets.

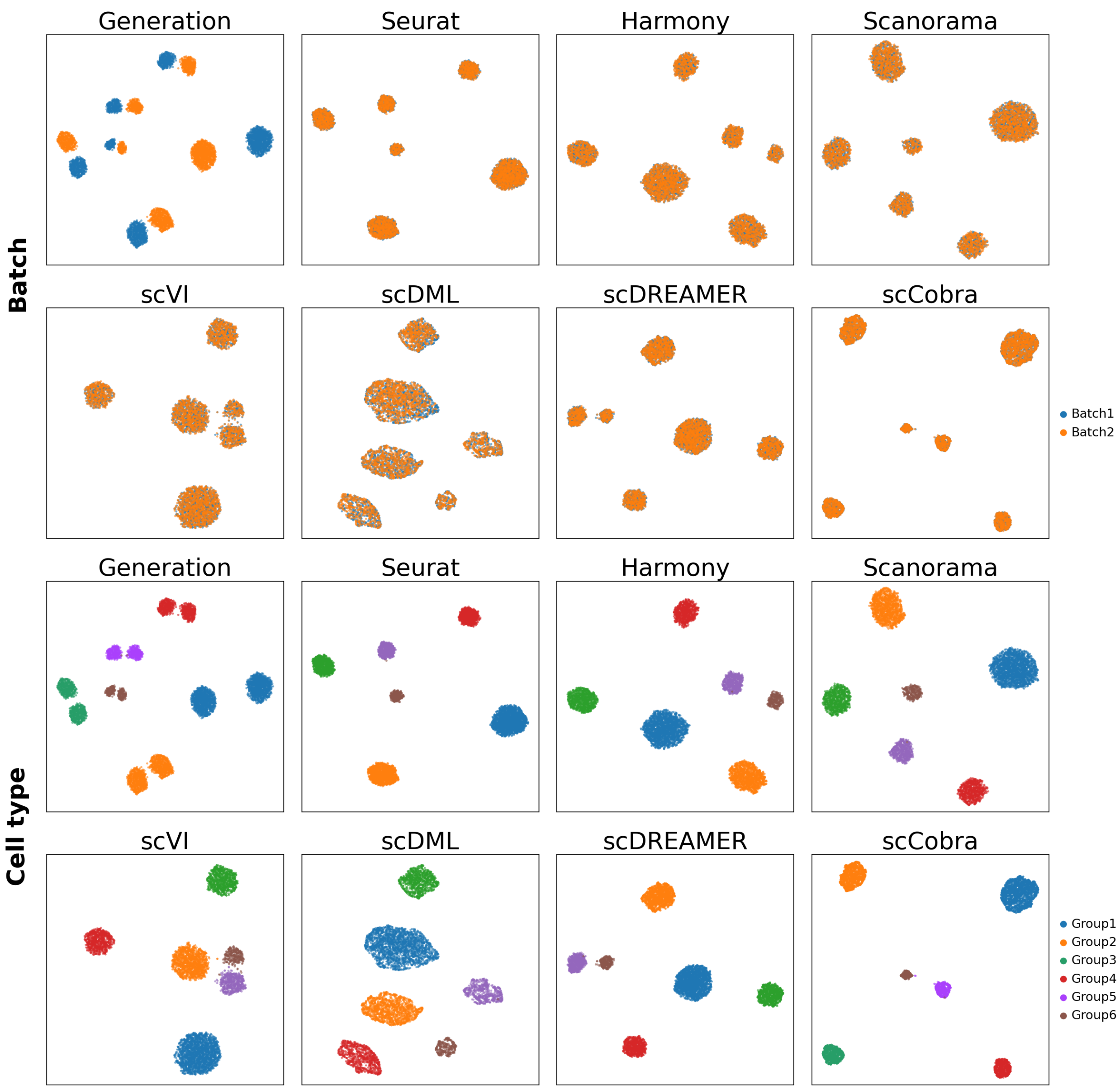

Fig. S10 scCobra enables simulation of scRNA-seq data with batch effects UMAP visualization comparing scCobra's performance against benchmarked methods (Seurat, Harmony, Scanorama, scVI, scDML and scDREAMER) on the scCobra-generated dataset (based on simulation dataset). The visualization is split into four rows: the top two rows show batch correction results, with different batches distinguished by color, and the bottom two rows present cell type aggregation, with each cell type assigned a unique color.

Supplementary Tables

Table 1: benchmarking table

Method	ARI	NMI	CellType ASW	Graph Connectivity	Batch ASW	Over-correction	Multi-omic integration	Label Transfer	Batch Generation	Original-space Correction
scCobra	**1/7**	**1/7**	**1/7**	**1/7**	4/7	2/7	**1/7**	✓	✓	✓
Seurat	6/7	6/7	4/7	4/7	2/7	5/7	**1/7**	✓	×	✓
Harmony	4/7	5/7	4/7	5/7	7/7	5/7	7/7	×	×	✓
Scanorama	5/7	3/7	2/7	6/7	**1/7**	**1/7***	6/7	×	×	✓
scVI	2/7	2/7	6/7	3/7	4/7	3/7	3/7	✓	×	✓
scDML	7/7	7/7	5/7	7/7	6/7	7/7	6/7	×	×	×
scDREAMER	3/7	4/7	3/7	2/7	2/7	3/7	4/7	×	×	×

*: Scanorama has the lowest over-correction score, but it tends toward under-correction.

✓: Native implementation. ×: No native implementation provided. 1/7: Ranked 1st out of 7 methods.

Table 2: Model architecture parameters

Module	Dimension
Encoder (E)	2000, 1024, 10
Decoder (D)	10, 2000
Fixed Encoder (E_f)	2000, 1024, 10
Domain Discriminator (Dis^d)	10, batch number
Cell level Contrastive head (Dis^c)	10, 10
Cluster level Contrastive head (Dis^l)	10, cluster number
Discriminator head (Dis^g)	10, 1

Table 3: Loss function parameters and trade-off parameters

Parameter	Value
λ	1
λ_{KL}	1
λ_{vae}	1
Temperature of Cell level Contrastive loss	0.1
Temperature of Cluster level Contrastive loss	0.5
