# Supplementary material for: scCobra allows contrastive cell embedding learning with domain adaptation for single cell data integration and harmonization
Source: Commun Biol. 2025 Feb 13;8:233. doi: 10.1038/s42003-025-07692-x (PMC11825689; doi:10.1038/s42003-025-07692-x)
Supplement: Supplementary file 2 — Description of Additional Supplementary Files [file 42003_2025_7692_MOESM2_ESM.pdf]

Description of Additional Supplementary Files

File name: Supplementary Data

Description: The source data behind the graphs in the paper.
