# Supplementary material for: scCobra allows contrastive cell embedding learning with domain adaptation for single cell data integration and harmonization
Source: Commun Biol. 2025 Feb 13;8:233. doi: 10.1038/s42003-025-07692-x (PMC11825689; doi:10.1038/s42003-025-07692-x)
Supplement: Supplementary file 4 — Reporting Summary [file 42003_2025_7692_MOESM4_ESM.pdf]

nature portfolio

Corresponding author(s): Jun Ding

Last updated by author(s): Jan 25, 2025

Reporting Summary

Nature Portfolio wishes to improve the reproducibility of the work that we publish. This form provides structure for consistency and transparency in reporting. For further information on Nature Portfolio policies, see our Editorial Policies and the Editorial Policy Checklist.

Statistics

For all statistical analyses, confirm that the following items are present in the figure legend, table legend, main text, or Methods section.

n/a	Confirmed	
☐	☒	The exact sample size (*n*) for each experimental group/condition, given as a discrete number and unit of measurement
☐	☒	A statement on whether measurements were taken from distinct samples or whether the same sample was measured repeatedly
☐	☒	The statistical test(s) used AND whether they are one- or two-sided<br>*Only common tests should be described solely by name; describe more complex techniques in the Methods section.*
☒	☐	A description of all covariates tested
☐	☒	A description of any assumptions or corrections, such as tests of normality and adjustment for multiple comparisons
☐	☒	A full description of the statistical parameters including central tendency (e.g. means) or other basic estimates (e.g. regression coefficient) AND variation (e.g. standard deviation) or associated estimates of uncertainty (e.g. confidence intervals)
☐	☒	For null hypothesis testing, the test statistic (e.g. *F*, *t*, *r*) with confidence intervals, effect sizes, degrees of freedom and *P* value noted<br>*Give P values as exact values whenever suitable.*
☒	☐	For Bayesian analysis, information on the choice of priors and Markov chain Monte Carlo settings
☒	☐	For hierarchical and complex designs, identification of the appropriate level for tests and full reporting of outcomes
☒	☐	Estimates of effect sizes (e.g. Cohen's *d*, Pearson's *r*), indicating how they were calculated

Our web collection on statistics for biologists contains articles on many of the points above.

Software and code

Policy information about availability of computer code

Data collection	No software was used for data collection.
Data analysis	The source code and tutorials of scCobra (software reported in this manuscript) are publicly available at https://github.com/mcgilldinglab/scCobra. scCobra is implemented using Python 3.9, installed via Anaconda (https://www.anaconda.com/), and Pytorch 1.12.1 (https://pytorch.org/).<br><br>Other tools used for downstream analysis include:<br>scikit-learn (v1.3.2) (https://scikit-learn.org/stable/)<br>SCANPY (v1.9.6) (https://scanpy.readthedocs.io/en/stable/)<br>Omicverse (v1.6.4) (https://omicverse.readthedocs.io/en/latest/index.html)<br>R (v4.3.0) (https://www.r-project.org/)<br>Scib (v1.1.5) (https://github.com/theislab/scib)<br><br>For benchmarking our integration performance, we used the following methods:<br>Seurat (https://satijalab.org/seurat/)<br>Scanorama (https://github.com/brianhie/scanorama)<br>Harmonypy (https://github.com/slowkow/harmonypy)<br>scVI (https://docs.scvi-tools.org/en/stable/user_guide/models/scvi.html)<br>TOSICA (https://github.com/JackieHanLab/TOSICA)<br>SCALEX (https://github.com/jsxlei/SCALEX)

scDML (https://github.com/eleozzr/scDML)
scDREAMER (https://github.com/Zafar-Lab/scDREAMER)

For manuscripts utilizing custom algorithms or software that are central to the research but not yet described in published literature, software must be made available to editors and reviewers. We strongly encourage code deposition in a community repository (e.g. GitHub). See the Nature Portfolio guidelines for submitting code & software for further information.

Data

Policy information about availability of data

All manuscripts must include a data availability statement. This statement should provide the following information, where applicable:

- Accession codes, unique identifiers, or web links for publicly available datasets
- A description of any restrictions on data availability
- For clinical datasets or third party data, please ensure that the statement adheres to our policy

In this study, we used three real scRNA-seq datasets to evaluate our method, similar to the datasets that were used in the scIB's method. The human lung atlas dataset, which includes 16 batches, 17 cell types, and over 32,000 cells. The pancreas dataset was composed of 14 cell types mixed across nine sequencing platforms, where each sample of InDrop-seq data was regarded as an independent batch, including 16382 cells with 19093 genes, this dataset was collected from Gene Expression Omnibus (GEO) (GSE81076, GSE85241, GSE86469, GSE84133, GSE81608). The third immune atlas dataset contains 10 batches with 16 cell types, including 33506 cells with 15148 genes. This dataset is available with the GEO accession number GSE115189, GSE128066, GSE94820 and https://support.10xgenomics.com/single-cell-gene-expression/datasets/3.0.0/pbmc_10k_v3. The simulation dataset was generated by the Splatter package. The dataset was composed of 12097 cells with 9979 genes, a mixture of seven cell types from six batches. In the over-correction evaluation, beyond Immune dataset, we also used Liver cancer data from GSE149614 and COVID-19 data from https://covid19cellatlas.org/. Multi-omic data are from https://support.10xgenomics.com/single-cell-gene- expression/datasets/3.0.0/pbmc_10k_v3 and https://support.10xgenomics.com/single-cell-atac/datasets/1.0.1/atac_v1_pbmc_10k. The MERFISH dataset has collected data on 64,373 cells with 155 genes, and the scRNA-seq dataset includes 30,370 cells with 21,030 genes. The datasets used for label transfer evaluation come from GSE83139, GSE114297 and GSE81547.

Research involving human participants, their data, or biological material

Policy information about studies with human participants or human data. See also policy information about sex, gender (identity/presentation), and sexual orientation and race, ethnicity and racism.

Reporting on sex and gender	N/A
Reporting on race, ethnicity, or other socially relevant groupings	N/A
Population characteristics	N/A
Recruitment	N/A
Ethics oversight	N/A

Note that full information on the approval of the study protocol must also be provided in the manuscript.

Field-specific reporting

Please select the one below that is the best fit for your research. If you are not sure, read the appropriate sections before making your selection.

☒ Life sciences ☐ Behavioural & social sciences ☐ Ecological, evolutionary & environmental sciences

For a reference copy of the document with all sections, see nature.com/documents/nr-reporting-summary-flat.pdf

Life sciences study design

All studies must disclose on these points even when the disclosure is negative.

Sample size	This study did not involve sample collection.
Data exclusions	With the single-cell RNA-sequencing datasets used in the study, we filtered out cells with more than 20% mitochondrial reads, which are indicative of poor-quality cells. We also removed genes not expressed by a minimum of 3 cells. These tasks were performed to remove low-quality cells and genes.
Replication	All computational experiments can be replicated using the code provided in our GitHub repository: https://github.com/mcgilldinglab/scCobra
Randomization	Randomization was not applicable to this study and therefore was not performed.
Blinding	Blinding is not applicable to method development.

Reporting for specific materials, systems and methods

We require information from authors about some types of materials, experimental systems and methods used in many studies. Here, indicate whether each material, system or method listed is relevant to your study. If you are not sure if a list item applies to your research, read the appropriate section before selecting a response.

Materials & experimental systems

n/a	Involved in the study
☒	☐ Antibodies
☒	☐ Eukaryotic cell lines
☒	☐ Palaeontology and archaeology
☒	☐ Animals and other organisms
☒	☐ Clinical data
☒	☐ Dual use research of concern
☒	☐ Plants

Methods

n/a	Involved in the study
☒	☐ ChIP-seq
☒	☐ Flow cytometry
☒	☐ MRI-based neuroimaging

Plants

Seed stocks	N/A
Novel plant genotypes	N/A
Authentication	N/A
